# Effect of Water-Induced and Physical Aging on Mechanical Properties of 3D Printed Elastomeric Polyurethane

**DOI:** 10.3390/polym14245496

**Published:** 2022-12-15

**Authors:** David Schwarz, Marek Pagáč, Josef Petruš, Stanislav Polzer

**Affiliations:** 1 Department of Applied Mechanics, Faculty of Mechanical Engineering, VSB-Technical University of Ostrava, 708 00 Ostrava, Czech Republic; 2 Department of Machining, Assembly and Mechanical Metrology, Faculty of Mechanical Engineering, VSB-Technical University of Ostrava, 708 00 Ostrava, Czech Republic; 3 Central European Institute of Technology, Brno University of Technology, Purkyňova 656/123, 612 00 Brno, Czech Republic; 4 Institute of Materials Chemistry, Faculty of Chemistry, Brno University of Technology, Purkyňova 118, 612 00 Brno, Czech Republic

**Keywords:** water aging, polyurethane, mechanical properties, physical aging

## Abstract

In this study, the effect of moisture on the elastic and failure properties of elastomeric polyurethane (EPU 40) 3D printed via Vat Photopolymerization was investigated. EPU 40 samples were printed, and uniaxial tensile tests were performed on Dry-fresh, Dry-aged (eight months aged), and after various times of being immersed in water (0–8 months). Elastic response, initial stiffness, failure strength, and failure elongation were analyzed. Besides, gravimetric analysis was performed to determine the increase in weight and thickness after water immersion. The elastic response was fitted by the Arruda-Boyce constitutive model. Results show that initial stiffness decreased after immersion (mean 6.8 MPa dry vs. 6.3 MPa immersed *p*-value 0.002). Contrary, the initial stiffness increased due to physical aging under a dry state from a mean 6.3 MPa to 6.9 MPa (*p* = 0.006). The same effect was observed for stiffness parameter *G* of the constitutive model, while the limit stretch parameter $\lambda_{L}$ was not affected by either aging. The 95% confidence intervals for strength and failure stretch were 5.27–9.48 MPa and 2.18–2.86, respectively, and were not affected either by immersion time or by physical aging. The median diffusion coefficient was 3.8·10−12 m^2/s. The immersion time has a significant effect only on stiffness, while oxidative aging has an inverse effect on the mechanical properties compared to water immersion. The transition process is completed within 24 h after immersion.

## 1. Introduction

Additive manufacturing is a rapidly emerging field that allows for the production of complex components and is one of the fastest-growing technologies. One of the latest 3D printing methods is Continuous Digital Light Processing–CDLP or CLIP (Continuous Liquid Interphase Printing–the marketing name of the Carbon company), which can print parts made of both plastic and hyper-elastic materials [1,2,3]. The ability of 3D printing techniques to create geometries impossible to obtain by other manufacturing methods allows the preparation of a new generation of products (e.g., Adidas shoes) while being relatively low cost per sample. In addition, single-part production makes it a perfect candidate for producing various patient-specific adjusted parts usable in wearable electronics [4,5], prostheses [6,7], implants [2,3,8], honeycomb structures with variable stiffness and damping [9] or lifestyle products [10] if they are made of a biocompatible material. Polyurethane (PU) based materials are very promising in this sense since they have excellent biocompatibility [11].

Several studies investigate the effect of water on the mechanical properties and mechanical behavior of printed polymers. Special research is focused on 4D printing and Shape Memory. The influence on the mechanical behavior of printed models is evident from the research. Manuscripts experimentally demonstrate the feasibility of using 3D printing to create a Shape Memory material with a precisely controlled shape change sequence that can be used to smoothly and successfully achieve specified material configurations. [12,13,14].

The next research is focused on the effect of water content on samples printed by Material Extrusion Fused Deposition Modeling technology. Three materials (nylon, a fiber-reinforced nylon composite, and polylactic acid (PLA)) exhibited different degrees of degradation, which was closely related to their unique molecular structure and hygroscopic nature. [15] As expected, the exposure to moisture at elevated temperatures resulted in an increased absorption rate.

Other studies also focused on the viscoelastic properties of elastomeric polyurethane EPU40 [3] and silicone [2], showing increasing hysteresis, stiffness, and strength with increasing loading speed.

The proper design of any part requires knowledge of the mechanical properties of the material the designed part is made of. The mechanical behavior needs to be investigated not only just after production but, more importantly, also in the typical environment the part will operate in. The effect of UV radiation, temperature, and moisture aging on molded PU, along with polymer oxidation, was studied previously. UV radiation has been shown to have a dramatic effect on mechanical properties [16,17]. Temperature accelerates the structural degradation of PU [18]. Temperature and moisture (together called hydrothermal aging, or HT) also showed a considerable effect on thermoplastic PU’s mechanical properties [16,19,20,21], but interestingly, it was not monotonous [19,20]. Thermal oxidative aging of polyurethane leads to a decrease in ductility and an increase in the modulus of elasticity [22].

One common drawback of performed studies is the limited applicability of published results for engineers since mechanical properties are typically reported as adopting the theory of linear elasticity, so the uniaxial tensile tests up to failure are performed, and Young modulus, strength, and failure strains are reported [16,19,20,21]. EPU is, however, by nature, hyperelastic material that undergoes large deformations and needs to be mathematically characterized by hyperelastic constitutive models in order to capture its behavior correctly [23]. Consequently, there is a lack of information about the stabilized (preconditioned) response of the molded PU [23], as well as a lack of information with respect to the physical and moisture-induced aging of 3D-printed PUs.

That is why we have focused on this part in this study and provided the stabilized mechanical response of PU samples 3D-printed by a Carbon 3D printer with respect to the time and time they were submerged in the water. This information is missing in the literature, yet it is critical for structural engineers who design cyclically loaded parts and products such as shoes or wearable electronics.

## 2. Materials and Methods

### 2.1. Sample 3D Printing

In this study, the CDLP method has been used for the production of samples made of PU (commercial name EPU 40) (Carbon, 2019). CDLP (also known as Continuous Liquid Interface Production or CLIP) works by projecting UV images generated by a digital light projector through an oxygen-permeable window in a UV-curable resin reservoir [24,25].

**Figure 1:**
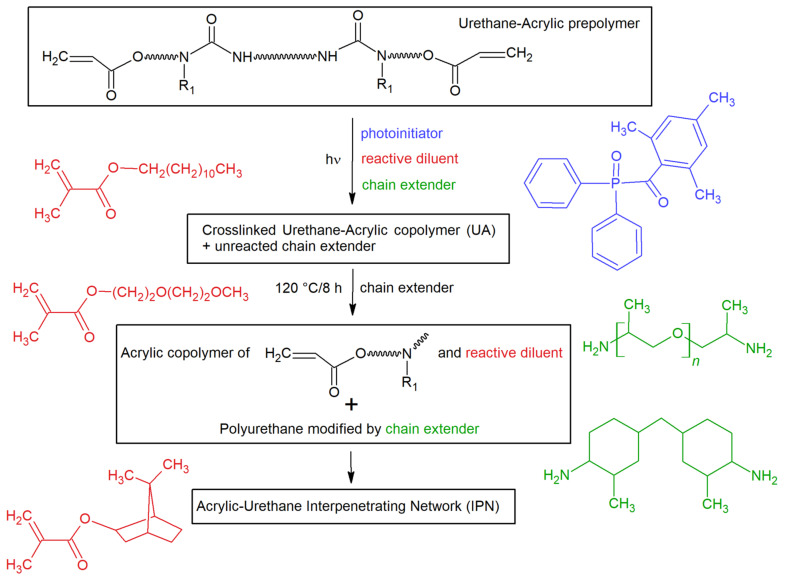
Scheme of the dual curing of EPU 40.

EPU 40 material is based on the interpenetrating network of the acrylic copolymer and modified polyurethane, whereas the structure of the interpenetrating network (IPN) formed during the dual curing process is responsible for the observed mechanical properties. Regarding the MSDS and TDS data, the formula of EPU40 consists of two parts: (i) Part A (η = 5100 cP at 25 °C) contains a mix of urethane-acrylic prepolymer, reactive diluents (lauryl methacrylate, 2-(2-methoxyethoxy)ethyl methacrylate, isobornyl methacrylate) and photoinitiator (diphenyl(2,4,6-trimethylbenzoyl)phosphine oxide); (ii) Part B (η = 110 cP at 25 °C) is comprised of a mix of chain extenders, namely 4,4′-methylenebis(2-methylcyclohexylamine) and poly(propylene glycol) bis(2-aminopropyl ether). Part A and Part B are mixed under the volume ratio 10:1. The formation of unique IPN structure during 3D printing and thermal annealing is schemed in Figure 1. In the first step, photocuring of acrylate-terminated PUR prepolymer and reactive diluents is initiated by the photoinitiator while chain extenders stay unreacted in the polymer network. In the second step, methacrylate units of the crosslinked urethane-acrylic copolymer are thermally cleaved, and newly formed reactive diisocyanates prepolymers react with diamine chain extenders which leads to the increase of both molecular weight and elongation in the order of 102% [25,26,27,28]. The proposed reaction mechanism is consistent with US Patent 9453142 [29] and that reported by Velankar, Pazos, and Cooper [26].

**Figure 2:**
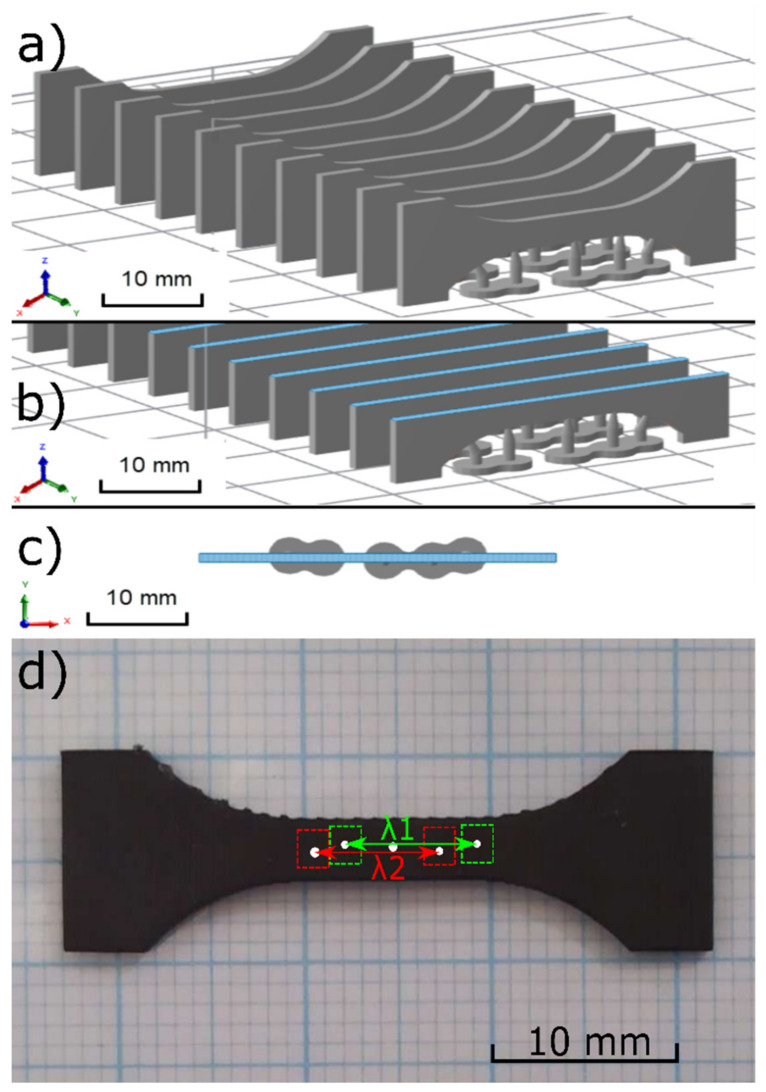
The Slicing process: (**a**) Isometric view, (**b**) isometric view for layer 130th, (**c**) top view for layer 130 (note: for illustration only), (**d**) Actual look of the 3D printed sample used for further analyses. Note small bulges in the neck area coming from the supports shown in Figure 2a.

The digital light projector has an in-plane pixel resolution of 75 μm/pixel and relies on the continuous motion of the build plate in the Z direction (upwards). This allows for a faster building time as the printer is not required to stop and separate the part from the building plate after each layer is produced [30]. A dog bone-shaped sample was created using SOLIDWORKS 2018 (Dassault Systemes SolidWorks, France), and EPU 40 samples were printed by a Carbon M2 3D Printer (Oerlikon, Germany). Fifty samples were prepared with layers oriented axially, as shown in Figure 2a. Sample orientation is a typical issue in 3D printing, but we showed previously that this method generates samples with isotropic properties [31].

The manufacturing of a dog bone-shaped specimen took 45 min, followed by sample conditioning at 120 °C for 8 h. The curing method was verified by modulated differential scanning calorimetry (see Figure S1 in Supplementary Data). The actual look of the samples can be seen in Figure 2d.

### 2.2. Mechanical Testing

The study was performed using a universal uniaxial test machine (M500-50CT). All 50 samples were included in the study. Five samples were tested (Dry-fresh) within a week after printing. The elastic response, failure stretch, and failure strength were obtained. The remaining 45 samples were placed in a dark container at a constant temperature of 22 °C to prevent thermal and UV-induced degradation. These samples were successively immersed in water for 5952, 1200, 696, 552, 168, 24, 1, and 0 (Dry-aged) hours prior to testing. Mechanical testing of all 40 samples was performed at once (within two days); thus, all samples were the same age (approximately 5952 h) at the time of testing. This setup of experiments allows separating the effect of water from other types of aging as samples vary only in the time they were immersed in water.

Dimensions of samples were measured via a digital caliper, which was clamped onto the machine. Uniaxial testing was then performed in the following manner: (1) The sample was preloaded to 0.2 N to make sure the sample was straight. (2) Six preconditioning cycles up to the estimated global stretch (calculated from clamp to clamp distance) of λ_Global_ = 1.9 were performed using a loading speed of 10 mm/min (≈loading rate of $7\cdot 10^{-3}\;s^{-1}$). The displacement limits were kept the same. (3) A new unloaded state was estimated. (4) Final cycle up to failure was performed.

The first Piola–Kirchhoff stress was estimated from actual force *F* and undeformed dimensions using the known formula:

(1) $$P=\frac{F}{w\cdot t},$$

where *w* stands for sample neck width and *t* is undeformed sample thickness. Strength is the stress at the failure point. The deformations were analyzed optically using digital image correlation (DIC) with the MERCURY RT system (Sobriety, Kuřim, Czech Republic). This is necessary because the dog bone shape is not deformed homogeneously, so the global stretch λ_Global is not the same as the local sample stretch λ in the central region where the stress is evaluated. It is noted that DIC was used for tracking the centroids of individual dots on the sample (see Figure 2d) rather than providing a strain field for which the sprayed pattern would be necessary. Out of that, the sample stretch was calculated as the average stretch observed between points 1 and 4(λ_1_) and 2 and 5(λ_2_), as shown in Figure 2d. The fifth dot was placed there for redundancy to ensure the analysis could be performed even if some of the dots lost their contrast due to cyclic stretching. Failure stretch was estimated as stretch corresponding to sample strength. Initial stiffness *E*_0_ was calculated as secant stiffness between stretches λ = 1 and λ = 1.05.

An Arruda-Boyce [32] constitutive model was fitted to each experimental curve in order to study mechanical response across the entire tested domain. The strain energy is defined as follows:

(2) $$W=G\left[\frac{1}{2}\left(I_{1}-3\right)+\frac{1}{20\lambda_{L}^{2}}\left(I_{1}^{2}-9\right)+\frac{11}{1050\lambda_{L}^{4}}\left(I_{1}^{3}-27\right)+\frac{19}{7000\lambda_{L}^{6}}\left(I_{1}^{4}-81\right)+\frac{519}{673,750\lambda_{L}^{8}}\left(I_{1}^{5}-243\right)\right]$$

where G stands for initial shear modulus, $I_{1}$ is the first invariant of the Right Cauchy Green deformation tensor, and $\lambda_{L}$ is the limit stretch at which the polymer chains are stretched. The stress-strain relationship can be obtained from Equation (2) using standard derivation described elsewhere [33]. The advantage of this model is in numerical stability and robustness in predicting other loading states, which makes it a perfect candidate for engineering computations. On the other hand, its disadvantage is the limited ability to capture the typical S-shape of the stress-strain curve of rubbers [34].

The effect of submerged time on sample strength, failure stretch $E_{0},\;G$ and $\lambda_{L}$, was tested statistically using the Kruskal–Wallis test and the test for equality of variances. The effect of aging was analyzed via the nonparametric Mann–Whitney test. The level of significance was chosen as 0.05, and it was decreased using Bonferroni correction when repeated tests were performed.

### 2.3. Water Uptake Measurement

The last 5 samples were not used for mechanical testing. Instead, they were subjected to gravimetric analysis in order to investigate water uptake when submerged. Our interest here was primarily motivated by a mechanical point of view. Therefore, we focused on mass uptake and the change of sample thickness over time, as those parameters may affect the mechanical properties of the material.

Samples were marked for clear identification, submerged in water, and put into a dark insulated chamber to ensure a constant room temperature of 22 °C. Their weight and thickness were measured repeatedly after 840, 168, 24, 12, 6, 1, and 0 h. Each sample was removed from the water, and its surface was dried with paper towels. Its thickness was measured using a digital caliper (CD-15APX, Mitutoyo, Japan), while its weight was measured using an electronic balance (ABS320-4N, Kern & Sohn GmbH, Balingen, Germany) with a resolution of 0.1 mg and an accuracy of 1 mg. Then the sample was put back into the water. The relative change in weight and thickness was recorded, excluding inter-sample variability. This was possible because samples were not tested (and destroyed), unlike those subjected to mechanical testing. We performed a statistical comparison of measured weight at individual times using the nonparametric sign test, with the null hypothesis stating that there is no difference in mass/thickness between the two compared times and the alternative hypothesis stating that the earlier time is associated with lower mass/thickness. It is noted that those tests were conducted to remove the inter-sample variability, which is an inherent part of the tests described in Section 2.2.

Diffusion coefficient *D* was expressed in the Fickian domain (where mass uptake vs. square root of time can be fitted by line) according to diffusion theory, similarly as previously mentioned [19,35]:

(3) $$D=\frac{\pi }{h}{\left(\frac{M_{t}}{M_{\infty }}\cdot \frac{t}{4}\right)}^{2}$$

where *h* represents actual time, and $M_{t}$ and $M_{\infty }$ stands for mass uptake in a given time and in equilibrium, respectively. Finally, *t* is the sample thickness.

### 2.4. Chemical Structure Characterisation

The chemical structure of EPU 40 after 3D printing (Dry-fresh), dry aging (Dry-aged 5952 h), and wet aging (Wet 5952 h) was identified by Fourier-transform infrared spectroscopy (FTIR, Bruker Tensor 27). FTIR spectra were recorded in the wavenumber range of 4000–650 cm^−1^ in attenuated-total-reflectance mode (ATR) using diamond crystal, a resolution of 4 cm^−1^, and 32 scans. The intensity of selected absorption bands was determined using Omnic software.

## 3. Results

**Figure 3:**
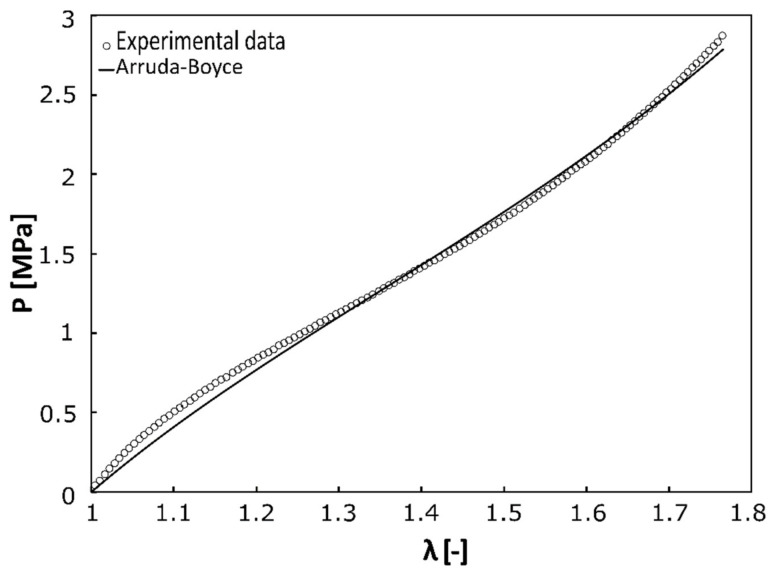
Typical experimental curve fitted by Arruda-Boyce model. This model somehow underestimates initial stiffness despite globally good fitting.

All samples were tested successfully. A global stretch of 1.9 resulted in a neck stretch of some 1.75 (see Figure 3). The Arruda-Boyce model was able to successfully fit measured responses with a median coefficient of determinacy R^2^ = 0.99 and worst fit of R^2^ = 0.96.

### 3.1. Effect of Aging on Mechanical Response

**Figure 4:**
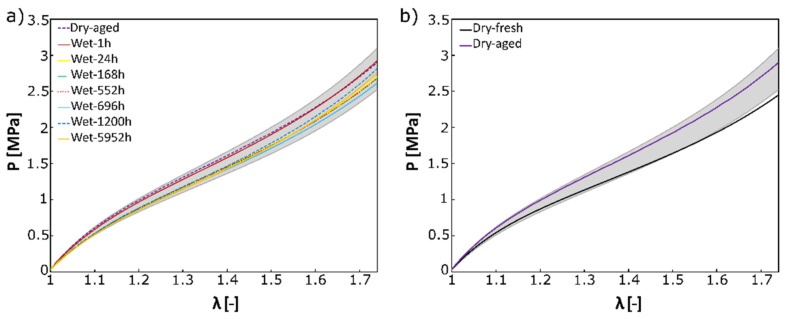
Mean stable elastic response EPU 40 up to λ = 1.75 shows (**a**) the effect of water aging (mean response of a sample of the same age but various submerged times is shown), and (**b**) the effect of physical aging dry samples only. The grey area represents all 40 curves constructed (samples immersed in water and Dry-aged samples). It is noted that the maximal stretch shown here is well below the failure stretch recorded in Table 2.

**Figure 5:**
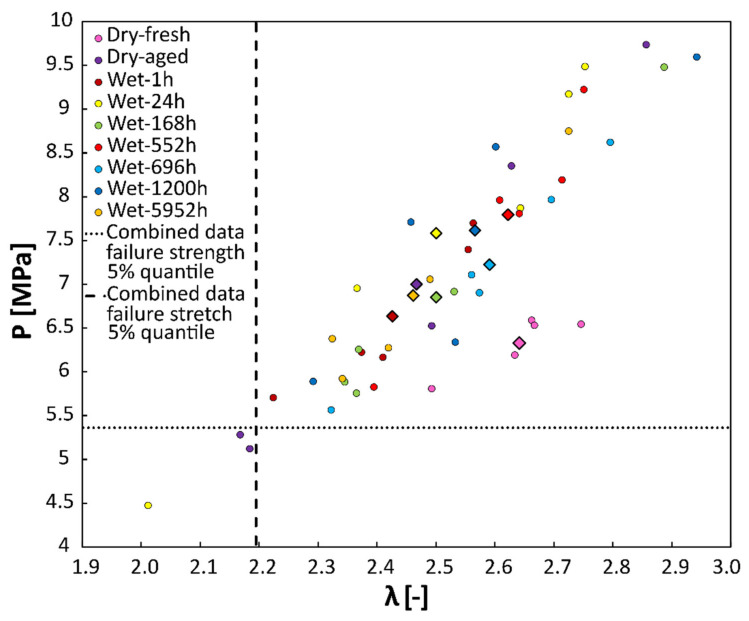
Strength vs. failure stretch for all samples. Colored circles represent individual measurements, while diamonds mark the mean value for each immersion time. The dotted line marks the 5% quantile of failure stretch for combined data, while the dashed line refers to the 5% quantile of strength also for combined data.

The mean stabilized elastic response did not show any significant dependence on the submerged time, as shown in Figure 4a. Similarly, the strength and failure stretch did not show any significant dependence on submerging time, as shown in Figure 5. Actual values for individual samples are reported in Table 1 and Table 2. *p*-values were 0.831 and 0.811 for strength and failure stretch, respectively.

The effect of physical aging is shown in Figure 4b. The mean stabilized elastic response of fresh samples (Dry-fresh) is 15% more compliant compared to Dry-aged samples, as shown in Figure 4b. Physical aging, however, affected neither strength (6.33 MPa vs. 7 MPa *p* = 0.5) nor failure stretch (2.64 vs. 2.47, *p* = 0.15), as shown in Table 1 and Table 2.

**Figure 6:**
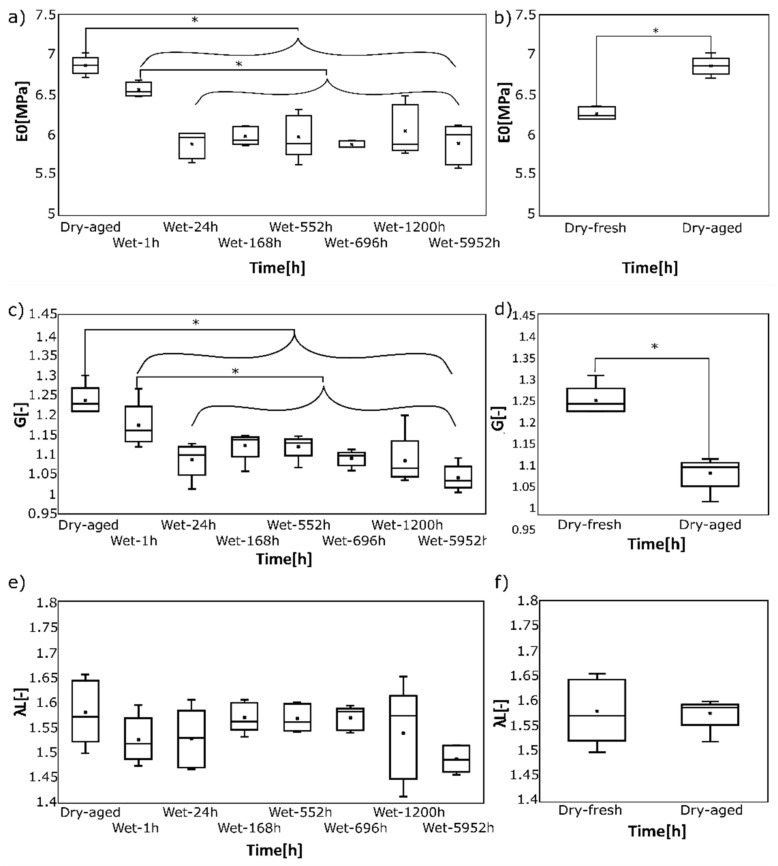
Effect of submerging time (**a**,**c**,**e**) and physical aging time (**b**,**d**,**f**) on mechanical properties. Asterix stands for statistically significant differences (**a**) Initial stiffness. Decrease of $E_{0}$ in the first 24 h is apparent, yet statistically significant different $E_{0}$ was confirmed only against the dried state. (**b**) Physical aging increases $E_{0}$ as an effect of oxidation and is statistically significant. (**c**) The shear modulus G of the Arruda-Boyce model decreases within the first 24 h in water. (**d**) Physical aging significantly decreases G. Limit polymer chain stretch $\lambda_{L}$ is not affected either by submerged time (**e**) or by physical aging (**f**).

Finally, the effect of submerging time on elastic properties is shown in Figure 6. Here, the values of *E*_0_ are statistically different from the Dry-aged state (*p* = 0.002), but no statistically significant difference was observed when the Dry-aged state was excluded, and the Bonferroni correction was included (*p* = 0.035). Also, a significant difference was observed between Dry-fresh and Dry-aged samples, as shown in Figure 6b. The median *E*_0_ was 6.9 MPa for Dry-aged vs. 6.3 MPa for Dry-fresh, respectively (*p* = 0.006). The effect of stiffness parameter *G* of the Arruda-Boyce model is similar. *G* is statistically different from the Dry-aged state and Wet-1h state (*p* = 0.001 and *p* = 0.005), but statistical significance vanishes for other submerging times when Bonferroni correction is applied. Physical aging reveals the decrease of the median of *G* by 13% from 1.23 MPa to 1.07 MPa (*p* < 0.001), as shown in Figure 6. Contrary, $\lambda_{L}$ values did not show a statistically significant difference in any conditions.

### 3.2. Water Uptake Measurement

**Figure 7:**
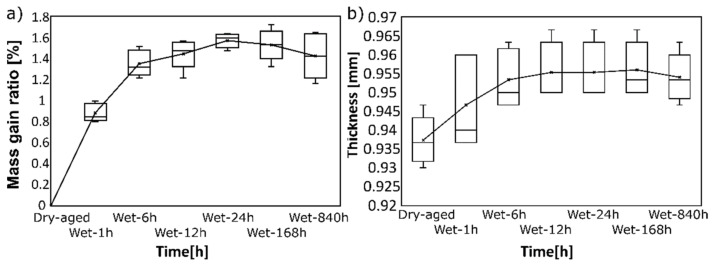
(**a**) Mass gain ratio and (**b**) absolute thickness change as a function of submerged time. Both quantities remain practically the same after 6 h of submerging.

Sample thickness and weight, in contrast, revealed significant dependence on submerging time, as shown in Figure 7. The sign test revealed a significant difference between 1 h and 6 h in water (both *p*-values = 0.031), but only the change after 1 h remains significant after the Bonferroni correction. The Fickian domain was estimated at h ≤ 2.5 (see Figure 7a), and the Diffusion coefficient median and 95% confidence interval was 3.8·10−12 m^2/s and $2.4\cdot 10^{-12}$ − 3.9·10−12 m^2/s, respectively. This is roughly by order higher compared to the sorption behavior of thermoplastic PUR (TPU D11T92EM) with a diffusion coefficient 1.2·10−11 m^2/s [19]. Based on the comparison of the absorption behavior of PUR-based IPN and TPU, it can be concluded that (i) a 3D network and the presence of chain entanglements eliminate the diffusion of water molecules into the EPU 40 structure more effectively, as evidenced by the lower diffusion coefficient, and (ii) only the surface diffusion occurs in the case of EPU 40 as evidenced from the lower duration of the equilibrium stage for EPU 40 (approx. 24 h), unlike TPU (170 h at 25 °C) [19].

### 3.3. Structure Characterisation

**Figure 8:**
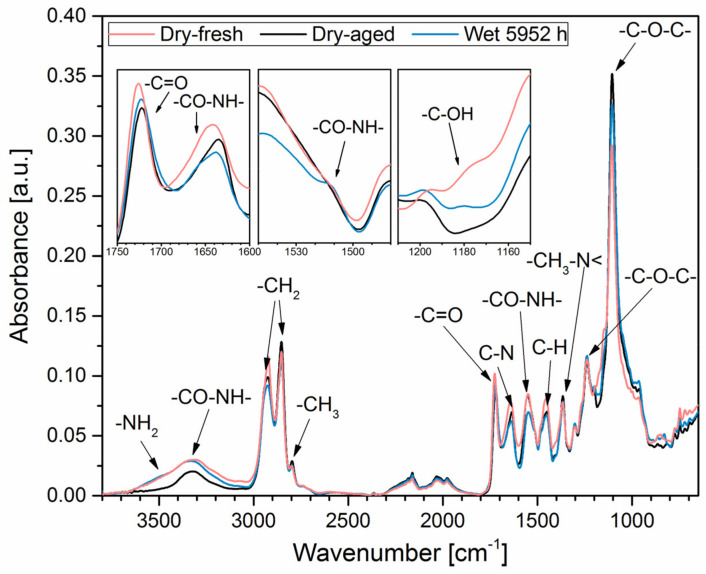
FTIR-ATR spectrum of Dry-aged (black curve), Dry-fresh (red curve), and Wet 5952 h (blue curve) samples of EPU 40.

The change of the chemical structure of EPU 40 after immersion in water was evidenced by FTIR-ATR. As shown in Figure 8, the most significant absorption bands characteristic of polyurethanes (PUR) were identified for both reference EPU 40 and EPU 40 immersed in water for 5952 h: (i) 3600–3200 cm^−1^ reflecting -NH- and -OH stretching vibrations; (ii) 2921 and 2853 cm^−1^ reflecting -CH2 asymmetric and symmetric stretching vibrations, respectively; (iii) 1722 cm^−1^–C=O stretching vibrations; (iv) 1648 cm^−1^–CO-NH vibrations; (v) 1550 cm^−1^–NH- deformation vibrations; (vi) 1451 cm^−1^–symmetric and asymmetric C-H deformations; (vii) 1367 cm^−1^–C-N deformation vibrations (CH3-N<); (viii) 1240 cm^−1^–C-O (C-O-C) stretching vibrations; (ix) 1109 cm^−1^–C-O asymmetric stretching vibrations. In addition, absorption bands in the wavenumber range of 1800–1600 cm^−1^ may also be characteristic of ester vibrations of (meth)acrylate comonomers representing a reactive diluent. Similarly, absorption bands in the wavenumber range of 3000–2800 cm^−1^ can be assigned to -CH2 vibrations of the chain extender in the PUR backbone.

Hydrolytic degradation of EPU 40 was also evaluated by the change of intensity of absorption bands corresponding to -CO-NH- urethane. The intensity of -CO-NH- absorption bands at 1640 cm^−1^ and 1550 cm^−1^ was related to the intensity of -CH2 absorption bands at 2925 cm^−1^ and 2855 cm^−1^, where the concentration of -CH2 groups is expected to be constant since -CH2 do not participate in the hydrolysis. According to data in Table 3, no significant surface hydrolysis of EPU 40 can be expected with respect to the negligible change of I1640/I2925, I1640/I2855, I1550/I2925, and I1550/I2855 after immersion in water, conditioning at room temperature, and 50% humidity.

## 4. Discussion

In this experimental study, we investigated the effect of physical and water aging on the stable elastic properties of EPU 40 obtained from 3D printing using the CLIP method [1]. Such knowledge is critical for engineers as the hyperelastic materials are known to exhibit significant softening during the first few cycles [23], and moisture is not prevented in most outdoor applications. Moreover, hyperelastic materials in engineering are almost exclusively used in a cyclic loading environment, so the information about the tensile test of the virgin sample is not sufficient for the design of products made of these materials.

### 4.1. Structure Changes Due to Water Aging

**Figure 9:**
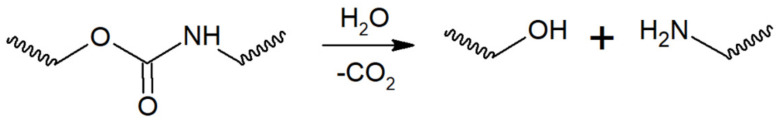
General scheme of PUR backbone hydrolysis.

We observed that water affects sample thickness by around 2%, which is negligible from an engineering point of view. A mass uptake of 1.6% was similar as reported for casted TPU [19] and further supports the idea that 3D printed EPU 40 is not porous [31]. If porous, the mass uptake is expected to be significantly higher compared to casted samples. A significant effect was observed only after the first few hours in the water and did not change over time. This is probably due to the structure of EPU 40 based on the interpenetrating network (IPN), which eliminates the chain mobility and, thus, the water diffusion towards a hydrolysable -CO-NH- urethane bond. The difference in FTIR spectra was observed in the wavenumber range of 3600–3000 cm^−1^, where weak absorption bands at ~3500 cm^−1^ and ~1180 cm^−1^ were observed for EPU 40 after immersion in water, probably due to a low extent of PUR backbone surface hydrolysis resulting in the formation of the terminal -NH2 and -OH groups, respectively (see the general mechanism in Figure 9). More significant trans- CO-NH bonds were observed for EPU 40 after immersion in water, probably due to a higher free volume between polymer chains.

The coefficient of diffusion was by an order lower compared to immersion in 70 °C water [18] which was expected since our water temperature was 22 °C. This can be explained by the glass transition temperature Tg~8 °C (see DSC thermogram in Figure S2 in Supplementary Data), which is slightly below the testing temperature of 22 °C as in accordance with data in the technical data sheet of the EPU40 supplier. Generally, the increase of (T-Tg) increment at T > Tg, the increase of diffusion coefficient due to enhanced chain mobility. The increase of *E*_0_ during long-term storage may be linked to several phenomena: (i) Possible physical aging given by chain mobility above Tg at room temperature may result in a more oriented structure with improved *E*_0_, and/or (ii) weak physical interactions participating in the increase of *E*_0_ (e.g., hydrogen bonding) due to moisture and low content of adsorbed water. Not reaching statistical significance for *E*_0_ and *G* for 12 and more hours of submerging may be caused by a low number of samples. Nevertheless, we showed that submerging for 24 h is sufficient for the waste majority of transient processes, and the response of 3D printed EPU 40 beyond it does not change significantly anymore.

### 4.2. Failure Properties Are Not Affected Neither by Physical nor by Water-Induced Aging

The effect of aging on failure properties was not observed at all. That is why we considered all samples as equal to an estimated 5% quantiles of both strength (5.27 MPa) and failure stretch (2.18), see Figure 5. These values can be further used by engineers as guaranteed limit values when designing their parts. It is underlined that reported values of failure stretch (both mean and guaranteed) of 3D printed EPU 40 are significantly lower (95% confidence interval (2.18–2.86)) than the failure stretch of casted PU [19,36] but also than those reported by the Carbon 3D company [27] where however much higher loading speed was used for the tests. We assume that the difference is caused by the preconditioning and lower loading speed rather than by the process of part creation.

The fact that the failure stretch in Table 2 is significantly above the upper stretch limit used to construct a stable elastic response (Figure 4) is natural, as we needed to ensure no sample tearing during preconditioning. The chosen range where a stable elastic response was measured is considered sufficient from an engineering point of view.

### 4.3. Hyperelastic Properties Are More Affected by Physical Than Water-Induced Aging

The stiffness parameters *E*_0_ and *G* of EPU 40 were observed to change during aging. In the case of *E*_0_, Physical aging causes a mild increase of around 10%. Contrary, we observed a median 15% decrease after 24 h in water (from 6.86 MPa to 5.9 MPa, see Figure 6). That is somehow lower than the 40% decrease reported elsewhere [36]. That study, however, created only virgin curves (not a stable preconditioned response). Though no details about EPU materials have been provided by Kanyanta and Ivankovic [36], a different chemical structure and architecture of EPU material can be expected with respect to the more significant change of *E*_0_. Dry room values of E_0_ depend dramatically on PUR composition, and reported values vary between around 3 MPa [7] and 41 MPa [19], so a quantitative comparison is not possible. Nevertheless, the decrease of *E*_0_ was several times greater than mass uptake. If the water were simply added to the sample in bulk, the same loading force would cause lower stress according to Equation (1) because of the change of sample dimensions. The decrease of *E*_0_ would be proportional to the dimension change (~4% in this case). The disproportional decrease of *E*_0_ could also be theoretically linked to the partial hydrolytic degradation of EPU 40; however, this was not proved by FTIR analysis (see Table 3) because of the IPN architecture of EPU 40 preventing the bulk water diffusion, as evident from a plateau of mass change and sample thickness reached at 24 h. Moreover, the hydrolytic degradation of EPU 40 is also suppressed by the chemical structure of PUR units. In consistency with the structure of EPU 40 reported in the literature [26,29], PUR units are composed of polyether soft segments (polypropylene glycol) and isophorone diisocyanate hard segments. It is well known that polyether polyols are more resistant to hydrolytic degradation compared to polyester polyols. Similarly, aromatic and cyclic diisocyanates are more hydrolytically stable than aliphatic diisocyanates (e.g., hexamethylene diisocyanate). Consequently, from the chemical point of view, EPU 40 represents hydrolytically stable material. In addition, chain extenders may also affect hydrolytic stability. Regarding the structure of chain extenders in the EPU 40 structure, they are based on the combination of polyether diamine (poly(propylene glycol) bis(2-aminopropyl ether)) and cyclic diamine 4,4’-methylenebis(2-methylcyclohexylamine. As mentioned above, polyether-based and cyclic diamines can be reasonably considered to be hydrolytically stable.

The stiffness parameter *G* of chosen hyperelastic model shows similar behavior compared to *E*_0__,_ which is expected as both quantities should, in theory, describe the sample’s initial stiffness. Theoretically, $E_{0}=3\cdot G$. Numerical comparison, however, shows clearly that this equation does not hold (see Figure 6), and the constitutive model predicts about 50% lower initial stiffness compared to experimental values. This indicates that this model does not capture initial stiffness perfectly, as shown in Figure 3. That is a known limitation of this model [34]. On the other hand, the most valuable feature in the constitutive modeling of hyperelastic materials is a stable model with the ability to predict loading states different from fitted ones rather than a perfect fit on uniaxial experimental results. Polynomial models are best in uniaxial curve fitting, yet they are not used anymore due to their instabilities and unrealistic predictions of other loading regimes. Finally, the fact, that $\lambda_{L}$ does not change at all means neither physical nor water-induced aging changes the shape of the tensile curve. This is because $\lambda_{L}$ is related to the point where sample stiffness starts to increase again.

In agreement with the results presented above, the hydrolytic degradation of EPU 40 is suppressed, and the decrease of *E*_0_ and *G* due to hydrolysis is not expected. The immediate changes caused by immersion in water are physical changes due to water absorption, where the penetration of water into the polymer network causes voids [20,37] and thus reduces *E*_0_ and *G* depending on the water uptake rate. In this case, water acts as a plasticizer reducing stiffness without cleavage of polymer chains [38]. Theoretically, hydrolysis of EPU 40 would result in a decrease of the network density, which could raise the water uptake with a further decrease of both stiffness and strength and an increase of elongation due to the rupture of chemical bonds. Nevertheless, stiffness, mass change, and thickness of samples reached a plateau at 24 h of immersion in water with no significant changes with a prolonged immersion period, which means that any possible ongoing changes in EPU 40 structures are below the resolution of this study and also below engineering significance.

### 4.4. Limitations

Despite encouraging results, some limitations of our study should be mentioned as well. First, it is a relatively low number of samples for each immersion time, so some statistically significant results could be missed. It is justified by the relatively high number of times at which the mechanical properties were evaluated. Moreover, we aimed for a stabilized response, so mechanical tests were much more time-consuming compared to traditional single tensile tests performed by others. The second limitation is the fact that we did not investigate the mass uptake and elastic properties at an elevated temperature.

## 5. Conclusions

In this study, we reported stable elastic properties of 3D printed EPU and showed that the immersion time has a significant effect only on values of initial stiffness *E*_0_ and constitutive model stiffness parameter *G*. They both decreased by around 15% with respect to the dried aged state. The physical aging test revealed stiffness increases by around 10% after eight months under dry conditions. The overall elastic response was around 15% stiffer after physical aging, while failure properties were insensitive to both physical and water aging. Any change in the investigated properties can be considered finished after a maximum of 24 h in the water under room temperature. The 5% quantiles of strength and failure stretch were 2.18 and 5.27 MPa, respectively. They can be used as limits for engineering applications together with *G* and $\lambda_{L}$ parameters of the Arruda-Boyce hyperelastic constitutive model.

## Figures and Tables

**Table 1:** Sample strength as a function of submerged time.

Submerged Time [h]	Dry-Fresh	Dry-Aged	1	24	168	552	696	1200	5952
Sample No	Strength [MPa]								
1	6.59	5.11	7.69	6.95	5.75	8.19	5.56	7.70	7.05
2	6.54	6.52	6.16	7.86	6.91	7.80	6.90	5.88	5.92
3	6.19	5.28	5.70	9.48	9.47	7.95	7.10	6.33	6.27
4	6.53	9.73	6.22	4.47	5.88	9.22	7.96	9.59	6.37
5	5.80	8.35	7.39	9.17	6.25	5.82	8.62	8.56	8.75
Mean	6.33	7.00	6.63	7.59	6.85	7.80	7.23	7.61	6.87
SD	0.3	1.79	0.77	1.8	1.37	1.1	1.04	1.38	1.01

**Table 2:** Failure stretch as a function of submerged time.

Submerged Time [h]	Dry-Fresh	Dry-Aged	1	24	168	552	696	1200	5952
Sample No	Failure Stretch [-]								
1	2.66	2.19	2.56	2.37	2.37	2.71	2.32	2.46	2.49
2	2.75	2.49	2.41	2.64	2.53	2.64	2.57	2.29	2.34
3	2.63	2.17	2.22	2.75	2.89	2.61	2.56	2.53	2.42
4	2.67	2.86	2.37	2.01	2.35	2.75	2.70	2.94	2.33
5	2.49	2.63	2.56	2.73	2.37	2.40	2.80	2.60	2.73
Mean	2.64	2.47	2.43	2.50	2.50	2.62	2.59	2.57	2.46
SD	0.08	0.26	0.13	0.28	0.21	0.12	0.16	0.22	0.15

**Table 3:** CO-NH-/CH2 ratio for EPU 40 Dry-aged and EPU 40 Wet 5952 h.

	I_1640_/I_2925_	I_1640_/I_2855_	I_1550_/I_2925_	I_1550_/I_2855_
EPU 40 Dry-fresh	0.664 ± 0.060	0.576 ± 0.019	0.758 ± 0.018	0.622 ± 0.051
EPU 40 Dry-aged	0.670 ± 0.060	0.522 ± 0.039	0.773 ± 0.080	0.613 ± 0.055
EPU 40 Wet 5952	0.647 ± 0.027	0.520 ± 0.028	0.737 ± 0.035	0.607 ± 0.025
	Δ (I_1640_/I_2925_) (%)	Δ (I_1640_/I_2925_) (%)	Δ I_1640_/I_2925_ (%)	Δ I_1550_/I_2855_ (%)
After Dry aging	+0.9	−9.4	+2.0	−1.4
After water exposure	−2.6	−9.7	−2.8	−2.4

## Data Availability

The raw data required to reproduce these findings are available to download from https://data.mendeley.com/datasets/wcwtjrkfsm (accessed on 2 September 2022).

## Supplementary Materials

The following supporting information can be downloaded at: https://www.mdpi.com/article/10.3390/polym14245496/s1, Figure S1: Modulated DSC thermogram of EPU40 after the dual-curing; temperature range from −50 to 220 °C, heating rate 2.5 °C/min., amplitude 0.3 °C, period 60 s, N2 atmosphere, 10–15 mg of sample was loaded in hermetically sealed aluminum pan, Figure S2: DSC thermogram of EPU40 after dual-curing; temperature range from −50 to 100 °C, heating rate 10 °C/min, N_2_ atmosphere, approx. 5 mg of sample loaded in the aluminum pan.
