# Supplementary material for: Effect of Water-Induced and Physical Aging on Mechanical Properties of 3D Printed Elastomeric Polyurethane
Source: Polymers (Basel). 2022 Dec 15;14(24):5496. doi: 10.3390/polym14245496 (PMC9783526; doi:10.3390/polym14245496)
Supplement: Supplementary file 1 [file polymers-14-05496-s001.zip › polymers-2052664-supplementary.pdf]

Supplementary data

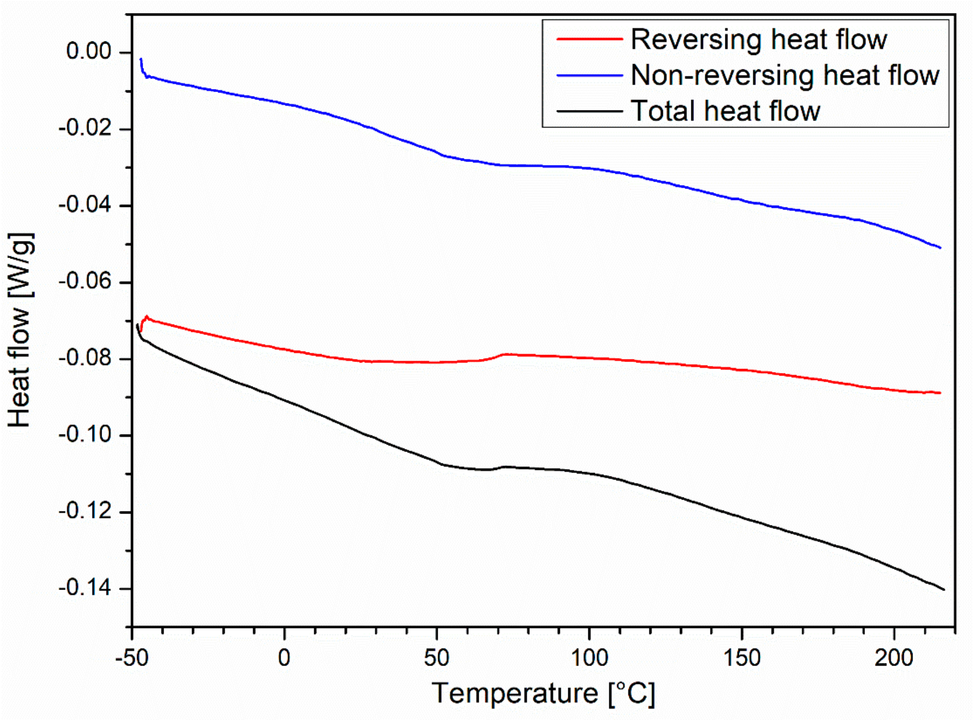

Figure S1. Modulated DSC thermogram of EPU40 after the dual-curing; temperature range from -50 to 220 °C, heating rate 2.5 °C/min., amplitude 0.3 °C, period 60 s, N_2 atmosphere, 10–15 mg of sample was loaded in hermetically sealed aluminum pan.

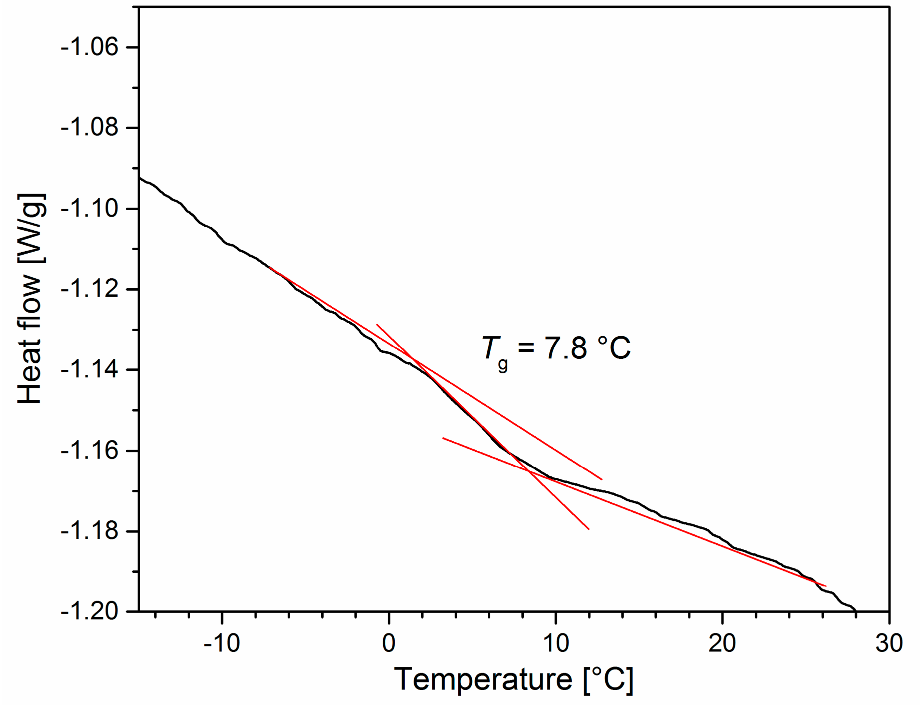

Figure S2. DSC thermogram of EPU40 after dual-curing; temperature range from -50 to 100 °C, heating rate 10 °C/min, N_2 atmosphere, approx. 5 mg of sample loaded in the aluminum pan.
